# Pediatric Patients with SARS-CoV-2 Infection: Clinical Characteristics in the United States from a Large Global Health Research Network

**DOI:** 10.7759/cureus.10413

**Published:** 2020-09-12

**Authors:** Ankita Desai, Alexandra Mills, Sarah Delozier, Claudia Cabrera Aviles, Amy Edwards, Sahera Dirajlal-Fargo, Grace McComsey

**Affiliations:** 1 Pediatric Infectious Diseases, University Hospitals Rainbow Babies and Children's Hospital, Cleveland, USA; 2 Center for Clinical Research, University Hospitals Cleveland Medical Center, Cleveland, USA

**Keywords:** covid-19, sars-cov-2, pediatric

## Abstract

Background

Few reports have been published on the clinical presentation of pediatric patients infected with severe acute respiratory syndrome coronavirus 2 (SARS-CoV-2). We aim to shed more light on the clinical presentation of pediatric patients infected with coronavirus disease 2019 (COVID-19), and also potential risk factors for more severe clinical case presentation.

Methods

We used a large global health research network to gather clinical data extracted from the electronic medical records of pediatric patients aged < 18 years with confirmed SARS-CoV-2 from January 1, 2020 to May 7, 2020. Clinical symptoms at presentation, hospitalization status, associated co-morbidities, and treatments received were reviewed.

Results

A total of 627 patients with COVID-19 diagnosis (334 were outpatient, 293 were inpatient) were included from a total of 20 organizations across the United States. The mean age of patients was seven years, 48% were females. Inpatients were younger than outpatients (mean age of 5.6 years vs 8.2 years, p<0.001). Sixty-one percent of patients in the inpatient group were < 5 years of age vs. 44% in the outpatient group. Amongst 293 inpatients, 90% (n=265) were non-severe and 10% (n=28) were classified as severe. The percentage of patients <5 years was higher in severe inpatients vs. non-severe (71% vs 60%.) Significantly more patients with a severe illness vs. non-severe illness had a history of co-morbidity including non-congenital heart disease (50% vs 11%, p<0.001) and disease of the respiratory system (86% vs 53%, p< 0.001).

Conclusion

Clinicians should closely monitor young children with underlying conditions and COVID-19, as they may be more likely to be hospitalized and have a higher severity of the disease.

## Introduction

In just the span of a few short months, we were notified of coronavirus disease 2019 (COVID-19), a severe respiratory illness, caused by the novel severe acute respiratory syndrome coronavirus 2 (SARS-CoV-2), with subsequent spread across the globe [1]. While we have gained a tremendous amount of knowledge regarding this virus, transmission, and clinical presentation, we still have much to learn [2]. Perhaps one of the most intriguing questions raised by this virus relates to the impact on children. We are yet to fully understand why children seem to be less susceptible to severe infection compared to adults [3].

In humans, coronaviruses mostly cause respiratory or gastrointestinal illness. Reported clinical manifestations range from the common cold to severe illness with severe acute respiratory distress syndrome and multi-organ failure [4].

Few reports have been published on the clinical presentation of pediatric patients infected with SARS-CoV-2 [1-2,4-7]. Recommendations for treatment of children with COVID-19 are largely extrapolated from adult studies [8]. With this report, we aim to shed more light on the clinical presentation of those pediatric patients infected with COVID-19, and also potential risk factors that may lead to more severe clinical case presentation. We looked at a large database, with specific attention to clinical symptoms at presentation, hospitalization status, associated co-morbidities, and treatments received for pediatric patients in the United States.

## Materials and methods

We used a large global health research network to gather clinical data extracted from the electronic medical records of pediatric patients aged less than 18 years with confirmed SARS-CoV-2 infection in 20 health care organizations in the United States from January 1, 2020 to May 7, 2020. We were able to differentiate inpatient (hospitalized) subjects from outpatients (those who were never hospitalized). In addition, inpatients were further characterized into severe progression, defined as requiring mechanical ventilation or death, or were otherwise classified as inpatients, non-severe. Clinical information was gathered for demographics, medical diagnoses prior to COVID-19 diagnosis, clinical symptoms and laboratory findings at COVID-19 presentation, and medication history at the time of COVID-19 diagnosis.

Data was analyzed using TriNetX (Cambridge, MA), a global federated health research network providing access to statistics on electronic medical records from approximately 53 million patients in 41 healthcare organizations. As a federated network, TriNetX received a waiver from Western Institutional Review Board. In order to prevent the possibility of patient re-identification the TriNetX platform takes several precautions. Amongst these, no individual patient records are available, and all data are reported as aggregates. TriNetX also rounds all patient counts from 1-10 as ≤ 10. We report this 1-10 value range as ≤ 10 and report the exact values of 0 as 0. In-platform analyses were conducted directly from the TriNetX platform, in which continuous data are presented as means and standard deviations, and inferential analyses were conducted using independent t-tests. Categorical data are presented as frequencies and percentages and inferential analyses were conducted using chi-square tests. Due to the obfuscation of patient numbers to keep the de-identification of data in the network, numbers are rounded thus creating differences within and between cohorts. All tests were two-tailed and p ≤ .05 considered significant.

## Results

Outpatients vs. inpatients

For this analysis, 627 patients with COVID-19 diagnosis (334 were outpatient, 293 were inpatient) were included from a total of 20 organizations across the United States (Table 1).

**Table 1:** Pediatric outpatient all vs. inpatient all and inpatient non-severe vs. inpatient severe Note: TriNetX software platform outputs all values 1:10 as ≤10. Continuous values are followed by the number of included patients in parenthesis. Due to the obfuscation of patient numbers to keep the de-identification of data in the network, numbers are rounded thus creating differences within and between cohorts. Mean ± standard deviations Number in parentheses reflect % of participants in group * represent p values <0.05

Variable	Outpatient all (N = 334)	Inpatient all (N = 293)	P-value	Inpatient non-severe (N = 265)	Inpatient severe (N = 28)	P-value
Age (yrs)	8.22 ± 6.78 (334)	5.6 ± 6.27 (293)	<.001	5.69 ± 6.3 (265)	4.68 ± 6 (28)	0.416
Sex (F)	162 (49)	137 (47)	0.662	119 (45)	18 (64)	0.051
Ethnicity						
Hispanic or Latino	64 (19)	17 (6)	<.001	15 (6)	≤10	0.999
Not Hispanic or Latino	68 (20)	68 (23)	0.388	55 (21)	13 (46)	0.002*
Unknown Ethnicity	202 (61)	191 (65)	0.224	178 (67)	13 (46)	0.028*
Race						
White	137 (41)	109 (37)	0.329	96 (36)	12 (46)	0.288
Black or African American	33 (10)	54 (18)	0.002*	46 (17)	8 (29)	0.231
Asian	≤10	≤10	-	≤10	0 (0)	-
American Indian or Alaskan Native	0 (0)	0 (0)	-	0 (0)	0 (0)	-
Native Hawaiian or other Pacific Islander	≤10	0 (0)	-	0 (0)	0 (0)	-
Unknown Race	157 (47)	144 (49)	0.592	137 (52)	7 (25)	<.001
Co-morbidities						
Congenital heart disease	0 (0)	0 (0)	-	0 (0)	0 (0)	-
Other forms of heart disease	20 (6)	41 (14)	0.001*	28 (11)	14 (50)	<.001
Cystic Fibrosis	(≤10)	(≤10)	-	≤10	≤10	-
Asthma	48 (14)	37 (13)	0.525	33 (12)	4 (14)	0.999
Premature lungs	0 (0)	0 (0)	-	0 (0)	0 (0)	-
Bronchopulmonary dysplasia originating in the perinatal period	(≤10)	(≤10)	-	≤10	≤10	-
Diseases of the Respiratory System	217 (65)	162 (55)	0.013*	140 (53)	24 (86)	0.001*
Neoplasms	28 (8)	28 (10)	0.607	23 (9)	5 (17)	0.906
Overweight, obesity	26 (8)	14 (5)	0.124	12 (5)	2 (7)	0.880
Diabetes insipidus	0 (0)	(≤10)	-	≤10	≤10	-
Diabetes mellitus	(≤10)	11 (4)	-	≤10	≤10	-
Endocrine, nutritional, and metabolic diseases	73 (22)	85 (29)	0.039*	67 (25)	21 (75)	<.001
Neuromuscular scoliosis	(≤10)	(≤10)	-	≤10	0 (0)	-
Congenital malformations, deformations, and chromosomal abnormalities	58 (17)	78 (27)	0.005*	59 (22)	20 (71)	<.001
Diseases of the nervous system	72 (22)	77 (26)	0.166	60 (23)	20 (71)	<.001
Diseases of the musculoskeletal system and connective system	88 (26)	66 (23)	0.267	52 (20)	15 (54)	<.001
Symptom at COVID-19 Diagnosis						-
Vomiting	11 (3)	13 (4)	0.456	≤10	≤10	
Pain in joint	0 (0)	(≤10)	-	≤10	0 (0)	-
Malaise and fatigue	(≤10)	14 (5)	-	≤10	≤10	-
Shortness of breath	(≤10)	(≤10)	-	≤10	0 (0)	-
Otalgia and effusion of ear	(≤10)	(≤10)	-	0 (0)	0 (0)	-
Nasal congestion	11 (3)	(≤10)	-	≤10	0 (0)	-
Myalgia	0 (0)	(≤10)	-	≤10	0 (0)	-
Muscle weakness (generalized)	0 (0)	(≤10)	-	≤10	0 (0)	-
Headache	(≤10)	(≤10)	-	≤10	0 (0)	-
Diarrhea, unspecified	(≤10)	20 (7)	-	16 (6)	4 (14)	0.211
Cough	35 (10)	40 (14)	0.272	33 (12)	7 (25)	0.121
Chills (without fever)	(≤10)	0 (0)	-	0 (0)	0 (0)	-
Anorexia	0 (0)	0 (0)	-	0 (0)	0 (0)	-
Acute pharyngitis	24 (7)	(≤10)	-	≤10	0 (0)	-
Fever of other and unknown origin	42 (13)	77 (26)	<.001	70 (26)	7 (25)	0.999
Dyspnea	14 (4)	43 (15)	<.001	29 (11)	14 (50)	<.001
Medications at COVID-19 Diagnosis						
Glucocorticoids	20 (6)	42 (14)	0.0005*	32 (12)	10 (36)	0.002*
Ibuprofen	16 (5)	43 (15)	<.0001	37 (14)	6 (21)	0.435
Ace inhibitors	0 (0)	(≤10)	-	≤10	≤10	-
Anticoagulants	(≤10)	26 (9)	-	21 (8)	5 (18)	0.159
Warfarin	0 (0)	(≤10)	-	≤10	0 (0)	-
Remdesivir	0 (0)	0 (0)	-	0 (0)	0 (0)	-
Hydroxychloroquine	0 (0)	(≤10)	-	≤10	≤10	-
Chloroquine	0 (0)	0 (0)	-	0 (0)	0 (0)	-
Azithromycin	(≤10)	13 (4)	-	11 (4)	2 (7)	0.804
Lopinavir/ritonavir	0 (0)	0 (0)	-	0 (0)	0 (0)	-
Tocilizumab	0 (0)	(≤10)	-	0 (0)	≤10	-

Pediatric patients ranged in age from <1 to 18 years, with a mean age of seven years, 48% were females. Race was unknown for 45% (n=282) of patients; of those known, 38% were white, 14% were African American, 2% were Asian. Ethnicity was known for 33% of patients and among those, 12% were Hispanic. 

Inpatients were significantly younger than outpatients (mean age of 5.6 years vs 8.2 years, p<0.001), specifically, 61% of patients in the inpatient group were < 5 years of age vs 44% in the outpatient group. There was no significant difference in sex between inpatient and outpatient groups (p=0.662). Among those for whom race was known, there was significantly more African American inpatients compared to outpatients (18% vs 10%, p=0.002), but no difference for white participants (p=0.329). Among patients for whom ethnicity was known, there were more Hispanic or Latino outpatients compared to inpatients (19% vs 6%, p< 0.001).

There was no significant difference between the groups for patients with a history of asthma (14% outpatient, vs 13% inpatient, p=0.524), neoplasms (8% outpatient vs 10% inpatient, p=0.607) or history of obesity (8% outpatient vs 5% inpatient, p=0.123). As shown in Table 1, patients hospitalized were more likely to have a history of endocrine/metabolic disease (22% outpatient vs 29% inpatient, p=0.039), congenital malformations or chromosomal abnormalities (17% outpatient vs 27% inpatient, p=0.005) as well as non-congenital heart disease (6% outpatient vs 14% inpatient, p<0.001).

Common symptoms recorded for outpatients at time of COVID-19 diagnosis included fever and cough in 13% and 10%, respectively. Other symptoms include dyspnea (4%), pharyngitis (7%), nasal congestion (3%), and vomiting (3%). Less than 3% of subjects had malaise/fatigue, headache, ear complaints, diarrhea, or chills. In contrast, 26% of inpatients had fever reported at time of diagnosis, 15% with dyspnea, 14% with cough, 7% with diarrhea, 5% with malaise/fatigue, and 4% with vomiting. Less than 3% of subjects reported nasal congestion, headache, pharyngitis, chills, or ear complaints. Significantly more inpatients had fever and dyspnea (p=0.001) at the time of COVID-19 presentation.

In terms of laboratory values, few outpatients had laboratory evaluation recorded. More values were recorded for inpatients. Several laboratory values were significantly more elevated in inpatients including: mean lactate dehydrogenase (LDH) (396 vs. 270 units/L, p=0.018), mean C reactive protein (CRP) (23.7 vs. 4.56 mg/L, p=0.044) and mean amylase (143 vs 38 U/L, p-0.045).

Less than 3% of outpatients were given azithromycin, but none were reported to have remdesivir, hydroxychloroquine/chloroquine, lopinavir/ritonavir, or tocilizumab. Among inpatients, 4% were given azithromycin, none were given remdesivir, and less than 3% received hydroxychloroquine. Tocilizumab was given to <10 inpatients and 9% of inpatients were anticoagulated. Significantly more inpatients received ibuprofen and glucocorticoids (p<0.001).

Inpatients non-severe vs. severe

Amongst 293 inpatients, 90% (n=265) were non-severe and 10% (n=28) were classified as severe (Table 1). Of the severe inpatients, 27 were mechanically ventilated and one patient died.

**Figure 1:**
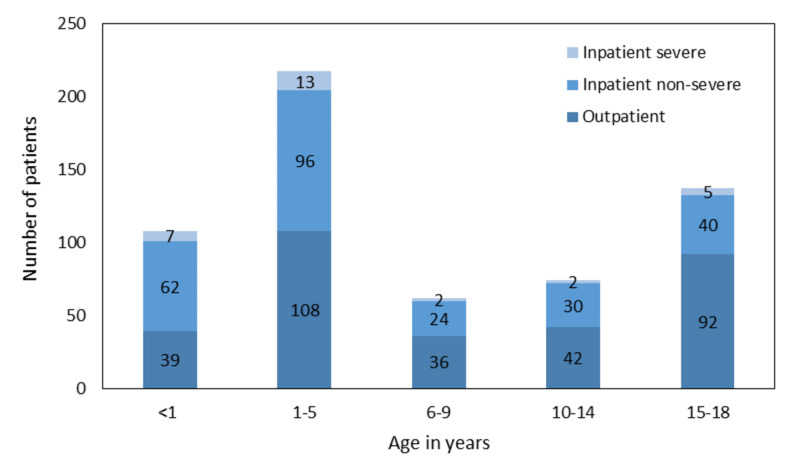
Hospitalization status and severity by age group Note: Numbers represent actual number of patients in each age group. Actual age is missing for about 15-20 patients in the cohort. This can be explained by one or few healthcare organizations contributing patients under 18 to the network but not disclosing actual age.

Overall there was no significant age difference, however, the percentage of patients <5 years was higher in severe inpatients vs. non-severe (71% vs 60%) (Figure 1).

Although not significantly different, there was a trend towards more females classified as being severe (64% severe vs 45% non-severe, p=0.051). Of those with known ethnicity, significantly more severe patients were not Hispanic or Latino (46% vs 21%, p=0.002). There was no significant differences for those with known race.

Significantly more patients with a severe illness had a history of co-morbidity including non-congenital heart disease (50% vs 11%, p<0.001), disease of the respiratory system (86% vs 53%, p< 0.001) metabolic disease/endocrine disease (75% vs 25%, p<0.001), congenital malformation or chromosomal abnormalities (71% vs 22%, p<0.001), and disease of the nervous system (71% vs 23%, p<0.001). Similar trends were seen among patients less than five years of age, more patients in the severe category had a history of co-morbidity (data not shown).

The severe patients were more likely to have dyspnea (p<0.001).

The severe inpatient group had significantly elevated prothrombin time (15.9 vs 13.6 seconds, p=0.028) and activated partial thromboplastin time (36.5 vs 29.2 seconds, p=0.018). Additionally, alanine aminotransferase and aspartate aminotransferase were also higher in the severe inpatient group compared to non-severe, 268 vs. 43.9 U/L (p=0.003) and 212 vs 55.6 U/L (p=0.008), respectively.

Azithromycin was given to 11 (4%) of the non-severe inpatients and two severe inpatients. Tocilizumab was only given to severe inpatients (n≤10). Steroids were more likely to be given to severe inpatients (36% vs 12%, p=0.002). Anticoagulation was recorded for 26 (9%) inpatients, 21 were non-severe, and five were categorized as severe.

## Discussion

At the time of submission, there was limited published data on clinical characteristics of pediatric patients with COVID-19 in the United States from large health care organizations. Our findings add to the current knowledge of clinical presentation and care of COVID-19 in children in the outpatient and inpatient settings.

In our analysis, a higher proportion of pediatric COVID-19 cases were hospitalized when compared to prior reports reported [5-6,9]. However, consistent with our current knowledge, more severe clinical presentations of COVID-19 were found in younger children under the age of five years requiring hospitalizations. Patients with an underlying condition were more likely to require hospitalization and have a severe presentation.

Global data suggest that there is a sex difference in mortality from COVID-19 [10]. In a preliminary analysis from the CDC, 57% of COVID-19 pediatric cases were males [5], and similarly slightly more males than females were affected in reported pediatric data from China [6]. In our study, there was no significant sex differences between patients requiring hospitalization, however, once hospitalized there was a trend for increased severity in females. This supports that sex differences are likely multi-factorial and may include sex hormone-influenced mechanisms as well as gender-based behavioral factors which may not become relevant until puberty.

Persons who are African American or black have higher mortality in the US, with an infection rate more than three-fold higher in predominantly black counties compared to predominantly white counties [11]. In our analysis, we found that there was significantly more African American inpatients compared to outpatients. Our study design does not allow us to adjust for comorbidities, however, we highlight that racial differences in COVID-19 disease severity which may be due to health care disparity extend to the pediatric population.

Outpatients reported the characteristic COVID-19 symptoms of fever and cough, however, more severe patients requiring hospitalization had fever and dyspnea. Very few patients presented with diarrhea or vomiting, unlike what has been reported for patients with Multisystem Inflammatory Syndrome in Children (MIS-C) [12]. Initial coagulopathy of COVID-19 in adult patients typically presents with elevation of D-dimer and fibrin degradation products, while abnormalities in prothrombin time and partial thromboplastin time are uncommon [13]. Our findings support the prevalence of activation of the coagulation cascade in the pediatric population with a severe presentation. This is significant as it is often predictive of a poor outcome or high mortality [14]. 

We found that few pediatric patients are receiving azithromycin and hydroxychloroquine, and those hospitalized with severe presentation are more likely to be given tocilizumab and anticoagulants.

This study has several strengths. To our knowledge, this is the first report from a large research network database with a focus on pediatric COVID-19 allowing us to have a more comprehensive picture of inpatient vs outpatient pediatric presentation. This study also has a number of limitations. Due to the nature of the database, we were unable to collect patient level data on specific outcomes and prognosis and duration of illness. We were unable to report on radiology information. We also were unable to report respiratory support needed such as nasal cannula, invasive or non-invasive ventilation, or on impact of any treatments given. We do not have information on type of diagnostic test used for confirmation of disease, whether nasopharyngeal swab vs. antibody testing vs. viral testing from other location (such as rectal swab). We were unable to report on source of transmission, whether travel related, community-acquired, or household source. Lastly, we do not have long-term follow up data on these patients. We do not know whether any of these patients had symptoms consistent with MIS-C after the initial COVID presentation.

## Conclusions

The number of COVID-19 cases continue to increase and our study suggests that clinicians should remain vigilant when monitoring young children with underlying conditions and COVID-19, as they may be more likely to be hospitalized and have a higher severity of disease. Studies on transmission of disease, clinical course, treatment, and prognosis are needed in pediatrics. Long term follow-up of these patients are needed to understand late manifestations of the disease, including MIS-C.
